# Resveratrol in Cancer Patients: From Bench to Bedside

**DOI:** 10.3390/ijms21082945

**Published:** 2020-04-22

**Authors:** Massimiliano Berretta, Alessia Bignucolo, Raffaele Di Francia, Francesco Comello, Gaetano Facchini, Manuela Ceccarelli, Rosario Vincenzo Iaffaioli, Vincenzo Quagliariello, Nicola Maurea

**Affiliations:** 1 Department of Medical Oncology-Centro di Riferimento Oncologico di Aviano (CRO), IRCCS, 33081 Aviano, Italy; 2 Experimental and Clinical Pharmacology-Centro di Riferimento Oncologico di Aviano (CRO), IRCCS, 33081 Aviano, Italy; alessia.bignucolo@cro.it (A.B.); francesco.comello@cro.it (F.C.); 3 Gruppo Oncologico Ricercatori Italiani, GORI-ONLUS, 33170 Pordenone (PN), Italy; rdifrancia@iapharmagen.com; 4 UOC Oncologia, ASL Napoli 2 Nord, P.O. “S.M. delle Grazie”, Pozzuoli-Ischia, 80078 Napoli, Italy; gafacchi@libero.it; 5 Department of Clinical and Experimental Medicine, Unit of Infectious Diseases, University of Catania, 95122 Catania, Italy; manuela.ceccarelli86@gmail.com; 6 Association for Multidisciplinary Studies in Oncology and Mediterranean Diet, Piazza Nicola Amore, 80138 Naples, Italy; eiaffaioli@libero.it; 7 Division of Cardiology, Istituto Nazionale Tumori- IRCCS- Fondazione G. Pascale, 80131 Napoli, Italy; quagliariello.enzo@gmail.com (V.Q.); n.maurea@istitutotumori.na.it (N.M.)

**Keywords:** resveratrol, cancer, inflammation, pharmacokinetic, pharmacodynamic, food-drug, personalized medicine, cytochrome P450, complementary medicines

## Abstract

Resveratrol (3,5,4′-trihydroxystilbene) is a natural phytoalexin that accumulates in several vegetables and fruits like nuts, grapes, apples, red fruits, black olives, capers, red rice as well as red wines. Being both an extremely reactive molecule and capable to interact with cytoplasmic and nuclear proteins in human cells, resveratrol has been studied over the years as complementary and alternative medicine (CAM) for the therapy of cancer, metabolic and cardiovascular diseases like myocardial ischemia, myocarditis, cardiac hypertrophy and heart failure. This review will describe the main biological targets, cardiovascular outcomes, physico-chemical and pharmacokinetic properties of resveratrol in preclinical and clinical models implementing its potential use in cancer patients.

## 1. Introduction

Resveratrol (3,5,4′-trihydroxy-*trans*-stilbene) is an antimicrobial and antioxidative compound (phytoalexin) with pleiotropic properties, naturally produced by plants and stored in many dietary sources like nuts, grapes, apples, red fruits, black olives, capers, red rice as well as red wines [1,2]. This interesting bioactive compound is well known as an antibacterial, antiviral and anti-inflammatory agent; however, in the last 10 years, researchers worldwide have exponentially increased the study on its biological properties, opening an unprecedented scientific interest in this field [3,4]. Notably, resveratrol is one of the most studied polyphenols that naturally accumulates in plant cells, and probably its presence characterizes the antioxidant and anti-inflammatory properties of a specific food [2]. The first studies concerning its chemical–physical structure demonstrated its interaction with the electrons scattered in reactive species of oxygen or other pro-oxidant components [5,6,7]. The worthwhile properties of resveratrol are, however, marked by a pharmacokinetic profile and by an unfavorable oral bioavailability, which requires a thorough analysis. To date, to the best of our knowledge, scientific papers and reviews summarizing multiple characteristics of resveratrol, its pharmacokinetic, pharmacodynamic profiles and potential drug interaction in oncology are lacking in literature. Therefore, this review aimed to describe the potential use of resveratrol in cancer management presenting the main biochemical pathways involved, issues and potential uses in cancer patients. Moreover, the involvement of resveratrol in cardioncology, its potential use for risk reduction of myocardial ischemia, myocarditis, cardiac hypertrophy as well as heart failure are also discussed. To endorse this review, we made a comprehensive search on PubMed, Web of Science and SciFinder. The search terms were “resveratrol” combined with “cardioncology” or “cancer” or “drug-food” or “cardiology” or “pharmacokinetic” or “pharmacodynamic”.

## 2. Physico-Chemical and Pharmacokinetic Properties of Resveratrol

**Figure 1:**
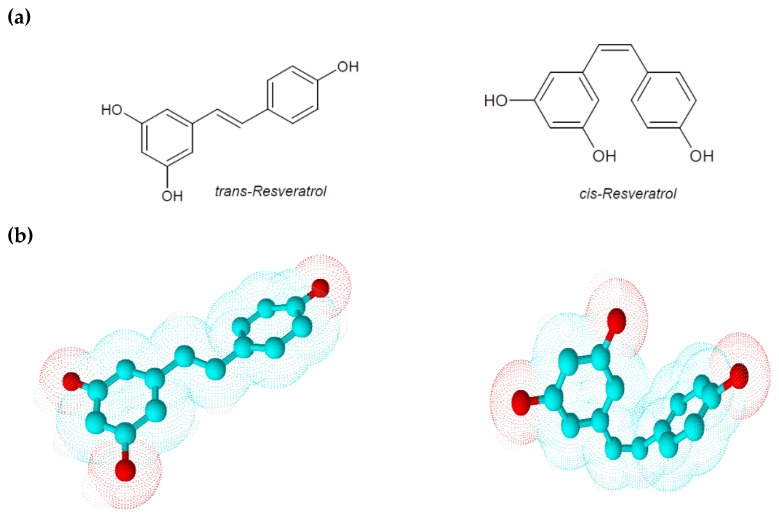
(**a**) Chemical structures of *trans*-resveratrol and *cis*-resveratrol; (**b**) 3D chemical structures of *trans*-resveratrol and *cis*-resveratrol.

Resveratrol is a low molecular weight phyto-polyphenol of 228 Da that appears as an off-white powder with lipophilic properties (octanol/water partition coefficient, log Kow = 3.1). Its melting point is between 253 and 255 °C, and its solubility in water is very low (3 mg/100 mL) [8]. The ethylene bridge due to a limited degree of freedom generates two geometric isomers: *cis*-resveratrol and *trans*-resveratrol (Figure 1) [5]. These isoforms co-exist in resveratrol extracts even if a greater biological activity and stability are attributed to *trans*-resveratrol. Comparing to the geometric isomers, the nearly planar structure of *trans*-resveratrol is less flexible than the non-planar structure of *cis*-resveratrol, but it possesses a lower repulsive energy that ensures it, albeit small, higher stability. Isomerization of *trans*-resveratrol to *cis*-resveratrol occurs via molecule breakdown when *trans*-resveratrol is exposed to UV radiation at a wavelength of 254 or 366 nm, to high pH conditions or in plants during fermentation processes [5,9]. In analyzing some physical–chemical aspects of resveratrol, Zupancic et al. showed how *trans*-resveratrol is rapidly degradable in high-pH solutions. In particular they demonstrated that *trans*-resveratrol exponentially decreases its stability and solubility at a pH ranging from 1.2 to 10 according to first-order kinetics and observing a higher degradation rate at 37 °C in a pH 7.4 buffer [9]. Since in humans the gastrointestinal tract pH ranges from 1.5 in the stomach to 8.5 in the intestine, this peculiarity could explain the low bioavailability of orally taken *trans*-resveratrol.

**Figure 2:**
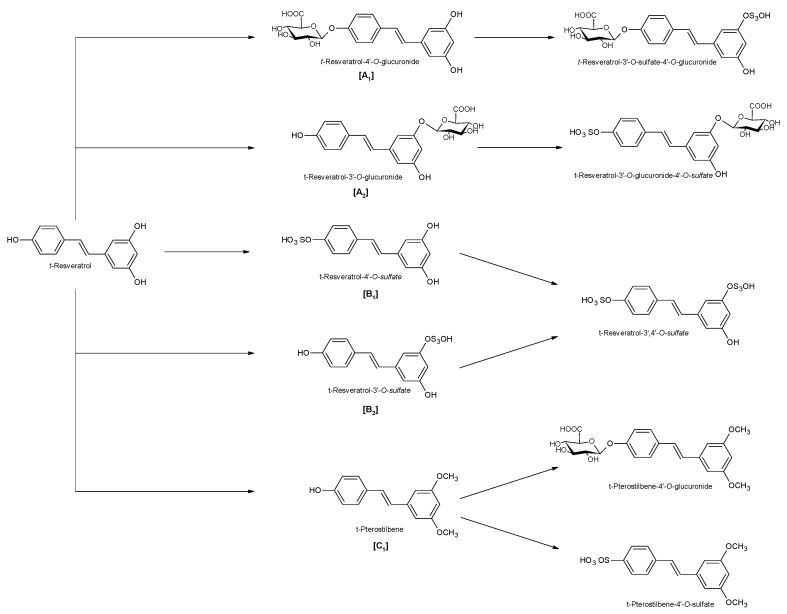
Phase II metabolism of *trans*-resveratrol. [A_1_]: 4′-glucuronic conjugation; [A_2_]: 3-glucuronic conjugation; [B_1_]: 4′-sulfate conjugation; [B_2_]: 3-sulfate conjugation; [C_1_]: 3,5-dimethyl conjugation.

**Figure 3:**
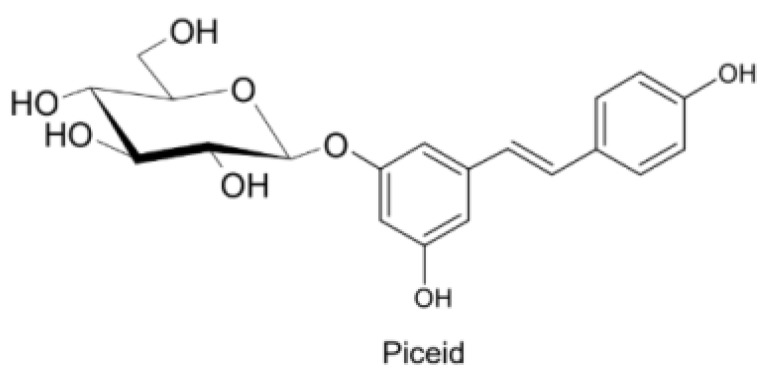
*Trans*-resveratrol-3-O-D-glycoside.

**Figure 4:**
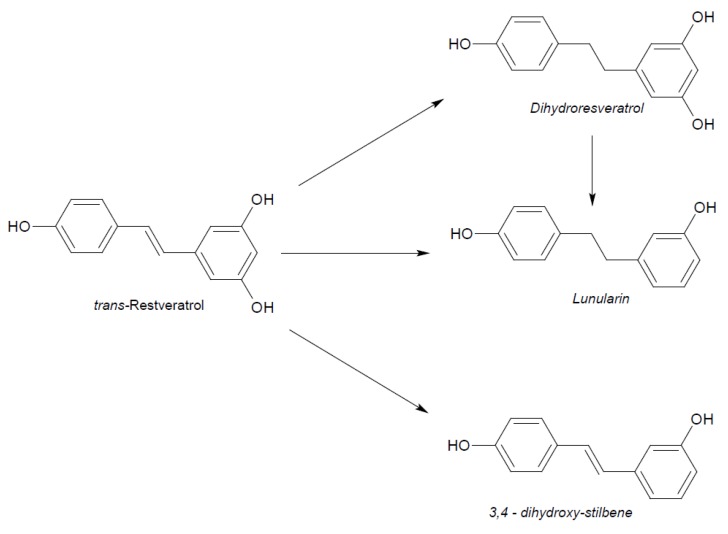
Example of gut microbiota biotransformation of *trans*-resveratrol.

**Figure 5:**
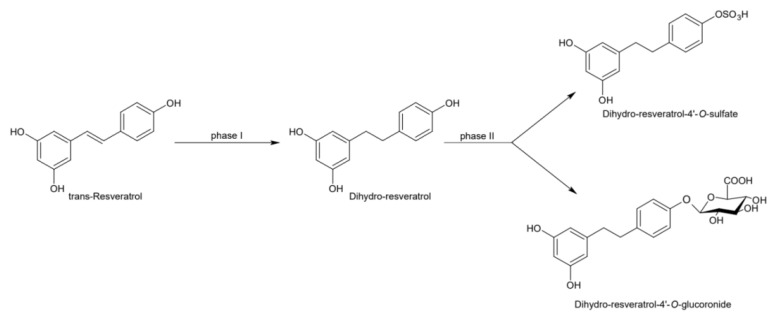
Microbial reduction of *trans*-resveratrol and conjugation of its main metabolite dihydro-resveratrol.

Naturally produced from phenylalanine in response to exogenous stimuli such as fungal infections from Botrytis cinerea, lesions or UV radiation, resveratrol is a phytoalexin present in numerous plants. It was first isolated around the 1940s from *Veratrum grandiflorum* roots, but the richest source of *trans*-resveratrol is known to be *Polygonum cuspidatum*, a plant widely used in traditional Chinese medicine [10,11]. Despite the numerous sources and their high variability in content, the use of *trans*-resveratrol is very limited due to its extensive metabolism (Figure 2) for which in vitro concentration does not reflect the actual concentration when considering administration in humans [12,13]. The substantial difference in plasma levels between in vitro and in vivo models partly is due to the rapid degradation and extensive metabolism to which resveratrol is exposed. When orally administered, resveratrol is rapidly absorbed via transepithelial diffusion as demonstrated by Walle et al. using ^14^C-labeled resveratrol. In this investigation, after oral administration of ^14^C resveratrol in healthy human volunteers, radioactivity was localized in epithelial cells of the gastrointestinal tract, and the absorption rate appeared to exceed 70% despite the rate of unchanged resveratrol in systemic circulation was extremely low [14]. This result was in line with what was found in other investigations using tritium-labeled resveratrol, in which absorption rates appeared between 70% and 90% [15,16]. Whilst *trans*-resveratrol seemed to passively diffuse and permeate the cell membrane, its glycosidic derivative *trans*-piceid (Figure 3) was seen to accumulate in cells and tissues to a lesser extent, as the absorption across the apical membrane seemed to be governed by the active Sodium-Glucose Transporter 1 (SGLT1) abundant in the small intestine. Only after passing the brush border membrane could *trans*-piceid then be hydrolyzed by cytosolic-β-glucosidase or by the membrane-bound lactase phlorizin-hydrolase releasing *trans*-resveratrol [17,18,19]. By contrast, *trans*-piceid, *trans*-resveratrol and relative conjugates resulted in substrates of the multidrug-related protein 2 (MPR2) and, to a lesser extent, the P-glycoprotein (MDR1), which were responsible for the apical efflux of these stilbenes. The ATP-binding cassette transporter (MRP3), placed in the basolateral membrane, was responsible for the diffusion of resveratrol and derivates into the bloodstream [17,18]. The low bioavailability of resveratrol also lies in the extremely organized crystalline structure that establishes its lipophilic nature. Indeed, its volume of distribution (Vd) after intravenous administration resulted about 1.8 L/kg, suggesting a considerable tissue distribution [14,16,20]. Moreover, *trans*-resveratrol and relative conjugates were found to be distributed mainly in the liver and kidneys, the primary sites of metabolism and excretion, but radioactivity of ^14^C-labeled resveratrol was observed also in the oral cavity, stomach and intestines suggesting a “resveratrol reservoir” of these tissues [14,20]. In accordance with previous investigations, Menet et al. confirmed, in an animal model, that after both single dose and sustained resveratrol intake, the distribution was higher in kidneys and the liver, whereas recently the pharmacokinetic analysis by Su and colleagues demonstrated that the major distribution of resveratrol, derivatives and metabolites occurred in the gastrointestinal tract after oral intake [21,22]. As mentioned above, resveratrol undergoes rapid and extensive metabolism, and several studies were conducted in order to identify resveratrol metabolites. It is alleged that resveratrol is exposed to phase II metabolism in order to reduce it lipophilicity [14,23]. The predominant molecular moieties of resveratrol circulating in plasma are its sulfate conjugate and glucuronide conjugate. Sulfation occurs in the liver and intestinal tract where resveratrol is a substrate of sulfotransferase (SULTs) isoforms SULT1A1, SULT1A3 and SULT1E. The major sulfate conjugates seem to be resveratrol-3-*O*-sulfate, resveratrol-4′-*O*-sulfate and dihydroresveratrol-sulfate [12,23,24]. Likewise, in the liver and intestine resveratrol undergoes glucuronidation carried out by uridine 5′-diphospho-glucuronosyl transferases (UGTs) [24,25,26]. Aumont and collaborators have proved with in vitro studies involving various UGT isoforms that glucuronic acid conjugation was regio- and stereoselective. *Cis*-resveratrol glucuronidation occurred 5 to 10 times faster than *trans*-resveratrol glucuronidation in both 3 and 4′ positions. Moreover, both in *trans* and *cis* isoforms conjugation, the 3-position was most promoted. From the analysis carried out with different UGT isoforms, it emerged that UGT1A9 and UGT1A10 exerted their activity toward both *cis*-resveratrol and *trans*-resveratrol, whereas UGT1A1 conjugated the 3-*O*-position of *trans*-resveratrol in a selective manner. *Cis*-resveratrol appeared to be selectively glucuronidated mainly by UGT1A6 and to a lesser extent by UGT2B7 and UGT2B15 [27]. Conjugation with glucuronid acid and with sulphate represents the rate-limiting step in the bioavailability of resveratrol, and such extensive phase II metabolism is responsible for the very low plasma concentration of free resveratrol. Several pharmacokinetic data in human healthy volunteers corroborated that, after oral administration, a higher concentration (Cmax) of free resveratrol occurred after about 1 h and was lower than the Cmax of resveratrol conjugates [14,26,28,29,30]. These data were consistent with the evidence, in animal models, that glucuronides and sulphate showed higher plasmatic concentrations than that of *trans*-resveratrol, confirming the rapid metabolism to which resveratrol undergoes [15,16,31,32]. Newsworthy, in both models a second plasma peak of resveratrol was highlighted, suggesting that resveratrol likely undergoes enteropathic recirculation. It could be assumed that both glucuronic and sulfate moieties undergo deconjugation, carried out by ubiquitous β-glucuronidases and sulfatases, releasing potentially active resveratrol [14,15,16,28,29,30,31,33,34]. The poor bioavailability of free *trans*-resveratrol depends not only on the high first-pass metabolism but also on its elevated protein binding. Albumin, indeed, seems to play a key role in establishing resveratrol–albumin complexes, thus concurring to 98.3% protein binding rate [35,36,37]. Interestingly the gut microbiota was another active major player in the metabolic pathway of resveratrol (Figure 4). Gut bacteria have proven to be skilled in improving the bioavailability of resveratrol by promoting its synthesis both starting from its precursors, such as piceid, and from its conjugates. In the gut lumen, indeed, all conjugates and metabolites may undergo hydrolysis and thus regenerate resveratrol. Once again, resveratrol may be a substrate of the intestinal bacteria, and it could be metabolized into its reduced form dihydro-resveratrol (DHR), which in turn could be further absorbed, both sulfate and glucuronic conjugated and excreted (Figure 5) [38,39,40]. Recent data revealed the gut microbiota to be a key player in the recovery of resveratrol. Notably, a study conducted on healthy volunteers revealed that the primary producers of dihydro-resveratrol were both *Slackia equolifaciens* and *Adlercreutzia equolifacens* strains. An in vitro testing, conducted with five different probiotic extracts, has also demonstrated that β-glucosidases, isolated from strains of *Lactobacillus acidophilus* and *Bifidobacteria infantis*, were able to catalyze the breakdown of piceid in its aglycone derivate [41]. This evidence, however, shall take into account the wide microbial diversity that may contribute to interindividual differences of resveratrol metabolism, which is the reason why further studies on the role of the gut microbiota with respect to resveratrol biotransformation are needed. Based on pharmacokinetic data collected after the intake of radio-labeled resveratrol in both human and animal models, it could be assumed that the preferential way of excretion of resveratrol and its metabolites is the renal one [14,16,20]. Radioactivity indeed was mostly recovered in urine and was very low along the gastrointestinal tract. Consistent with these findings, several other investigations remarked the renal excretion to be the major way of elimination of resveratrol [22,25,26,28,30,42].

## 3. Pharmacodynamic Properties of Resveratrol

Resveratrol is a nutraceutical phytoalexin that exerts its activity towards various disorders, despite the fact that its real mechanisms have not been fully understood yet and still remain a major challenge. Resveratrol has a good safety profile and has a wide spectrum of targets despite its employment as an active drug remaining limited due to its low bioavailability [43]. We will briefly describe the main mechanisms of action, so far known, of resveratrol to then deepen its benefits in cancer and cardiovascular diseases (Table 1). Resveratrol has antioxidant properties that ensure it an adjuvant role in all pathologies based on an excess of reactive oxygen species (ROS) production such as tumors, inflammation, cardiovascular disease (CVD), type 2 diabetes mellitus (T2DM) and neurodegenerative diseases. Its capability in contrasting low-density lipoprotein (LDL) oxidation via copper chelation due to its peculiar chemical structure was previously demonstrated [44,45,46]. Resveratrol was also reported to influence ROS levels both via free-radical scavenging and via upregulation of antioxidant enzymes like superoxide dismutase (SOD), glutathione peroxidase and catalase, which protects the cell from ROS damage, through different mechanisms [47]. Resveratrol, indeed, demonstrated to downregulate Hypoxia-Inducible Factor (HIF-1α), a protein widely expressed in cells undergoing hypoxia conditions, thus suppressing glycolytic metabolism [47]. For its structure, this stilbene was able to undergo a redox reaction acting as a quinone reductase and receive electrons from free radicals. A direct inhibition of quinine reductase 2 (QR2) by resveratrol was found to protect cells from the oxidative process via induction of antioxidant enzyme activity [48]. Lastly, data highlighted the capability of stilbene to activate p38-mitogen-activated protein kinase (p38-MAPK) signaling and suppress the extracellular signal-regulated kinases (ERK1/2) pathway responsible for increasing ROS levels [49,50,51,52,53,54]. Two of the main targets of resveratrol are known to be cyclooxygenase isoforms (COX-1 and COX-2), which metabolize arachidonic acid to generate prostaglandins (PG) and thromboxanes (Tx) both implicated in inflammatory response. The interaction of resveratrol with COX-1 and COX-2 leads to pro-inflammatory factors suppression [55,56]. Notably the inhibition of COX-1 in the arachidonic acid pathway, via suppression of thromboxanes TxA2, was found to be responsible for the anti-thrombotic activity along with the blockade of the mitogen-activated protein kinase (MAPK) signaling and the enhancement of the nitric oxide/cGMP pathway [57,58]. Moreover, data revealed that resveratrol exerts its effect toward lipooxygenase inhibiting the TxB2, a stable derivative of TxA2, involved in platelet aggregation and inflammatory processes [59]. Conversely, its inhibition of both inducible and constitutive COX-2 in the vascular endothelial cells was less selective compared to COX-1, leading to prostacyclin blockade and thus contrasting its vasodilatory and antiplatelet activities [58]. Specifically, the in vivo vasorelaxant activity of resveratrol was attributed to the capability to trigger the Ca^2+^-activated potassium (K_Ca_) channels and to stimulate both endothelium nitric oxide synthase (eNOS) and inducible nitric oxide synthase (iNOS) expression [60,61]. The vasorelaxing action of resveratrol is also carried out through the inhibition of both L-type Ca^2+^ channel and Ca^2+^ calmodulin cyclic nucleotide phosphodiesterase 1C (PDE1C) and the consequent decrease of contractile response of vascular smooth muscle cells (VSMCs). Resveratrol leads to lower contractile strength via eNOS induction and via limiting the intracellular Ca^2+^ release in VSMC, which reflects hypertension control [62]. Another potential target of resveratrol, with which it exerts anti-inflammatory activity, is represented by stimulation of Sirtuin-1 (Sirt-1) that impairs the inflammatory signal cascade of toll-like receptor 4/nuclear factor k-light-chain enhancer of activated B cells/signal transducer and activator transcription (TLR4/NF-κB/STAT), thus reducing the production of cytokines such as tumor necrosis factor α (TNF-α), interleukin-6 (IL-6), interleukin 1β (IL-1β) and monocyte chemoattractant protein-1 (MCP-1) or macrophage/mast cell-derived factors [63]. Particularly, resveratrol and its analogues were able to improve serum level of Sirt-1 with consequent preservation of endothelial homeostasis through the inhibition of TLR4/NF-κB/STAT pathways and related pro-inflammatory cytokines [63,64]. High levels of Sirt-1 were found to influence the expression of endothelial nitric oxide synthase (eNOS) responsible for producing nitric oxide (NO), a key player in the increase of vascular perfusion [60,61]. Several studies reported the capability of resveratrol in increasing the serum levels of adiponectin. Considering that this adipocyte-specific protein is significantly reduced in models of obesity and insulin resistance, and that its reduction is remarkably related to an increased risk of atherosclerosis, it is plausible that resveratrol could exert anti-atherosclerotic effects by increasing adiponectin serum levels [62,65,66,67]. High levels of adiponectin were indeed correlated with the inhibition of NF-κB signal and thus with the reduction of pro-inflammatory factors, preventing monocyte adhesion on vascular endothelium, the first process for atherosclerotic plaque formation [65]. Chronic low-grade inflammation, the formation of atherosclerotic plaques and platelet aggregation are, together with hypertension and reduced sensitivity to glucose, essential conditions for the development of the so-called metabolic syndrome that resveratrol has been shown to improve. Cardiovascular diseases (CVDs) are very often accompanied by obesity and impaired glucose metabolism. It was documented that resveratrol administration is able to decrease the plasma levels of glucose and glycated hemoglobin (HbA1c). The probable mechanism underlying the decrease of these markers was the activation of peroxisome proliferator-activated receptor gamma coactivator 1-alpha (PGC-1α) via Sirt-1 and the derived mitochondria biogenesis regulation. Insulin resistance indeed was found to be correlated to decremented PGC-1α activity [68,69,70]. Moreover, the positive effect of resveratrol on glucose levels was expressed through the activation of adenosine 5′ monophosphate-activated protein kinase (AMPK), a kinase closely interlinked to mitochondrial functions and metabolism and, thus, of type 2 diabetes mellitus (T2DM) development [68,69,70]. The abovementioned inhibition of NF-κB signaling by resveratrol was also considered a plausible anti-diabetic mechanism. The NF-κB pathway, indeed, was upregulated in T2DM, and the consequent IL-6 production led to cell insulin resistance. This condition resulted in both chronic high glucose plasma levels and high glycation products. These latter trigger the expression of glycation end-product receptors that, in turn, stimulate the NF-κB signaling cascade [71,72,73,74]. In recent years, resveratrol has arisen interest for its antitumor activity, and the underlying mechanisms are multiple and still under analysis. It demonstrated a double capability to both protect normal cells from radiation/chemotherapy damage and coadjuvate tumor cell suppression. Resveratrol was documented to interact with the complex tumor microenvironment network, a precondition for tumor growth, progression and metastasis. Particularly it was seen to play a key role in the apoptotic signaling of the tumor cell through direct stimulation of caspases cascade and the inhibition of the anti-apoptotic pathways such as PTEN/PI3K/AKT, Sirt-1, AMPK/YAP, NF-κB and STAT3 that contribute to tumor cell immortality [75,76,77,78,79,80]. The anti-proliferative property of resveratrol occurs with the enhancement of p21 and thus p53 activity. P21 is a strong inhibitor of Cyclin-Dependent Kinases and is able to link to the CDK2–CDK4 complex, responsible for the cell cycle progression from G1 to S phase. Such a bond allows p53 to definitely block the cell replication phase [81,82,83]. It emerged that resveratrol is able to induce the tumor cell cycle blockade also via inhibition of other intracellular signals such as Wnt/β-catenin, Hippo-YAP and Hedgehog that synergistically concur to tumor development, invasion and metastasis [54,84,85,86]. These pathways are also implicated in epithelial mesenchymal transition (EMT), a process conferring epithelial cells with mesenchymal characteristics necessary for tumor invasion. Furthermore, data demonstrated that the inhibition of PI3K/AKT, Hippo-YAP, activating protein (AP-1), Hedgehog and in particular HIF-1α contributed to reduce angiogenesis of new blood vessels required for tumor growth [50,51,52,54,87,88].

## 4. Resveratrol in Cardio-Oncology

As outlined above, several foods are naturally rich in resveratrol, like nuts, grapes, apples, red fruits, black olives, capers, brown or red rice and others [89]. Notably, they are very common in the Mediterranean diet which has demonstrated to have significant anti-inflammatory, anti-obesity and cardioprotective properties [90]. Being an extremely reactive molecule as well as capable to interact both with cytoplasmic and nuclear proteins [89,90] in human cells, resveratrol has been studied over the years as complementary and alternative medicine (CAM) for the therapy of cardiovascular diseases like myocardial ischemia [90,91], myocarditis [92], cardiac hypertrophy and heart failure [93,94]. The main mechanisms underlying its beneficial effects are based on its anti-oxidative and anti-inflammatory properties [89] as well as its improvements of calcium homeostasis in cardiomyocytes and reduction of cardiac fibrosis [95].

Preclinical and updated clinical evidence on the effects of resveratrol in cardiology is presented, with interesting implications in the cardio-oncological field, considering that some cardiac targets share common tumor targets, tightening on its possible use as a cardioprotective agent in cardio-oncology. Resveratrol has demonstrated to significantly reduce the oxidation of low-density lipoprotein (Ox-LDL), which is the key initiation point of atherosclerosis as well as a source of survival and resistance in breast, colon and prostate cancer cells through the involvement of β-catenin, cMyc, NF-κB, STAT1, STAT3 and other oncogenes [96,97,98]. Moreover, resveratrol has shown to reduce the risk of thrombosis through inhibition of platelet aggregation in preclinical models [99], Considering that venous thromboembolism is a major health problem among cancer patients [100], associated with high rates of mortality and morbidity, the possible use of resveratrol in this category of patients could be interesting.

Other data indicating the cardioprotective nature of resveratrol include the risk of atherogenesis. In preclinical studies, oral administration of resveratrol at 4 mg/kg/day reduced significantly the proliferation of smooth muscle cells, hyperplasia of endothelial vessels as well as the secretion of pro-inflammatory cytokines involved in the atherogenesis process [101]. Notably, another cardiovascular outcome associated with the administration of resveratrol in preclinical studies is the reduction of intimal proliferation index (ratio of intimal to (intimal + medial) area) of 45–50% compared to control animals with a significant reduction of α-actin expression in intima [102]. The main mediators of these effects are p53, heat shock protein HSP27, quinone reductases as well as nitric oxide [102]. Considering that several cancer therapies like androgen deprivation therapy are conceived to indirectly contribute to the development of hyperglycemia, dyslipidemia and metabolic syndrome, which are well-established causal risk factors of atherosclerosis [103,104], the use of resveratrol in this category of patients could be a therapeutic window of interest in the cardio-oncological field.

Another critical side effect seen in cancer patients is anthracycline-induced cardiotoxicity [105,106]. As recently summarized, cardiac event rates on doxorubicin therapy are 7%, 18% and 65% at cumulative doses of 150, 350 and 550 mg/m^2^, respectively [107]. Anthracycline chemotherapy was associated with an adjusted hazard ratio of 1.26 for development of congestive cardiac failure in 43,000 women (aged 66–70 years) with breast cancer over a median period of 53 months [107]. It is known that the mechanisms of doxorubicin-induced cardiotoxicity are mainly mediated by the involvement of oxidative stress as well as several pro-inflammatory mediators [108,109]. Resveratrol combined with doxorubicin significantly reduces the cardiotoxic phenomena in preclinical trials with the involvement of E2F1/AMPKα2 and E2F1/mTORC1 pathways [110]. Notably, resveratrol reduces apoptosis and necrosis of cardiac cells and improves calcium homeostasis, reducing also cytokine and collagen fibers, leading to a decrease of fibrosis in the myocardium [111]. The main mediators of these effects are the upregulation of Sirt-1, the reduction of transforming growth factor-beta and pSMAD3/SMAD3 [111]. In another recent study, co-administration of doxorubicin and resveratrol in mouse led to a reduction of both cardiac p38 MAPK activation and hypertrophy in response to Ang II-induced hypertension [112].

Clinical trials with resveratrol administration and cardiovascular outcomes are also described. First, resveratrol orally administered at 270 mg month in obese patients enhanced the endothelial functions with a significant increase of flow-mediated dilatation of the brachial artery [113]. Second, a similar study demonstrated that resveratrol improved levels of glucose and triglycerides [114]. Another trial involving patients with stable coronary artery disease demonstrated that resveratrol at 10 mg per day for 3 months improved left ventricle diastolic functions [115], and in a comparable study, double doses for 2 months led to a reduction of b-type natriuretic peptide (BNP) in patients with angina pectoris, demonstrating cardioprotective effects [116]. At present there are similar ongoing clinical trials based on the oral administration of resveratrol in patients suffering from heart failure with strong clinical expectations such as the improvement of the left ventricular ejection fraction, the reduction of inflammatory markers and indicators of myocardial damage [94]. The overall picture described herein places resveratrol as a potential cardioprotective tool in non-cancer as well as in cancer patients.

## 5. Metabolic Effect and Cancer–Drug Interactions

Recently, resveratrol has been shown to implement antineoplastic activities due to repression of neoplastic carcinogens by inhibiting phase I enzymes and synergistically by induction of phase II metabolic enzymes [117]. Resveratrol has demonstrated to interact with several antioxidant and anti-inflammatory agents. It supports an anticancer environment, through the inhibition/activation of the metabolic phase I enzymes, and induction/conjugation of phase II enzymes [118,119]. However, resveratrol supplementation would result in less activation of procarcinogens such as benzo-pyrenes. Phase I metabolism of xenobiotics like pharmaceutical drugs, phytochemicals, environmental pollutants and other endogenous compounds like hormones is accomplished mainly by the cytochrome P450 (CYP) family enzymes (Table 2).

It has been shown that resveratrol in preclinical studies inhibits the activity of CYP1A enzymes [120,121], and in particular it inactivates CYP1A2 in a microsomal mechanism-based assay. Nonetheless, resveratrol metabolites were likely responsible for the observed inhibitory activity [122]. A subsequent study found that a metabolite called RS3 did not inhibit CYP1A2 in cell cultures [123]. Another metabolite called piceatannol has been shown to inhibit CYP1A activity to an extent similar to that of resveratrol in rat hepatic microsomes and could affect its interactions with CYP1A enzymes. Since resveratrol induced CYP1A2 activity (evaluated in preclinical studies), the evidence from in vitro models demonstrated the inhibition of CYP1A1 and CYP1A2 by resveratrol. In an early clinical phase I study, in half the participants at 1 g daily resveratrol, 100 mg caffeine was added for 4 weeks, and the results were indicative of CYP1A2 induction [122,123]. The observed difference between this clinical observation and preclinical in vitro studies could be attributed to the indirect assessment of CYP1A2 activity, or to resveratrol metabolism. Since CYP1B1 is involved in the metabolism of catechol estrogen and the formation of a toxicologically active metabolite, 4-hydroxyestradiol, the inhibition of CYP1B1 is an attractive target for hormonally driven cancers such as breast. It is documented that 5 μmol/L resveratrol was able to reduce the formation 17b-estradiol through the inhibition of CYP1B1 in human mammary epithelial cells [124]. In overweight and obese postmenopausal women, 1 g/daily resveratrol for 12 weeks also had a favourable effect on estrogen metabolism [125]. Supplementation diet containing resveratrol for 12 weeks evidenced high anticoagulant levels by warfarin in a murine model, suggesting the inhibition of CYP2C9 [126]. As monitored in humans, a dose of 1 g daily resveratrol for 4 weeks was shown to inhibit CYP2C9 by 2.71-fold using losartan as a probe drug [122].

Resveratrol has demonstrated moderate inhibition of CYP2C19 in microsome models and the human recombinant form [121,132]. In cell line models, inhibition of CYP2D6 by resveratrol had low significance (50% inhibitory concentration IC_50_ of 87.9 mol/L) for resveratrol and its metabolites [127]. In humans administered 1 g/day resveratrol for 4 weeks, however, CYP2D6 activity decreased by 1.7-fold (dextromethorphan as probe), and warnings are suggested in combined treatments with tamoxifen [122,126]. Therefore, when considering resveratrol supplementation, it is necessary to predict potential interactions with CYP3A4 to ensure the safety of patients receiving chemotherapeutics. A pharmacokinetic study in 12 healthy males was conducted to determine the effect of resveratrol pretreatment on the pharmacokinetics of carbamazepine and on the CYP3A4 enzyme activity. Compared to controls, a single 500 mg dose of resveratrol administered once daily for 10 days prior to a single dose of carbamazepine 200 mg significantly increased maximal drug concentration (by 46.2%), area under the curve (by 37.1%) and half-life (by 22.8%) of carbamazepine and significantly decreased apparent oral clearance (by 33.1%) and apparent volume of distribution (by 19.3%). However, the time to reach maximum drug concentration and elimination rate constant had not significantly changed. Additionally, carbamazepine metabolite/parent ratios of C_max_ and AUC (Area Under the Curve) had also significantly decreased [128]. Several experiments strongly support an induction effect of resveratrol of several phase II enzymes including glutathione S-transferase (GST), nicotinamide hydrogenase (NADH), UDP-glucuronosyl transferase, (UGT) and catechol-O-methyl transferase (COMT). Resveratrol may facilitate the removal of carcinogens from the body. With regard to the association between GST induction and reduced cancer risk, in humans 1 g/day of resveratrol for 4 weeks induced GST activity among individuals, but the overall effect was not significant [122,129]. The induction of NQO1 gene, encoding for NAD(P)H dehydrogenase (quinine) enzyme, by resveratrol could have significant implications on breast cancer prevention. Estrogens were transformed into catechols and further oxidized into ortho-quinones, which react with DNA to form adducts [130]. By inducing NQO1, resveratrol may facilitate the reduction in semi-quinones to catechols and subsequent inactivation by COMT [130,131]. In a clinical trial, 1 g/day resveratrol for 4 weeks significantly increased bilirubin clearance among subjects with low baseline UGT1A1 activity; however, resveratrol intervention had a minimal effect on overall bilirubin clearance [122]. Nonetheless, evidence of nutrient–drug interactions suggests that this mechanism may still contribute to resveratrol’s overall anticancer properties. This evidence also shows that resveratrol interactions with drug metabolism are significant enough to warrant further investigation before its clinical recommendation. Although in vitro studies provide a controlled environment for precise quantification of resveratrol effect on phase I and II metabolism, they fail to capture the activity of resveratrol metabolites, which may have important clinical effects. Provided with the limited evidence available, we have attempted to consider the activity of resveratrol’s sulfated and glucuronidated metabolites to evaluate the translatability of certain animal models to humans.

## 6. Resveratrol and Cancer Evidence

**Figure 6:**
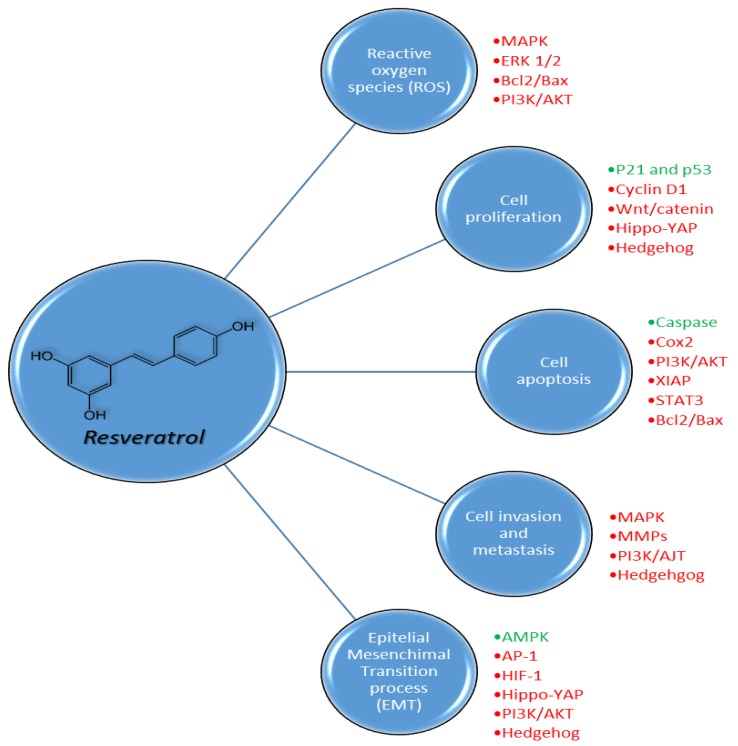
Pleiotropic biochemical effects of resveratrol on reactive oxygen species (ROS), cell proliferation, cell apoptosis, cell invasion and metastasis and epithelial mesenchymal transition processes in cancer cells through the activation (green) or inhibition (red) of different pathways.

With almost 10 million deaths per year worldwide, cancer is a major public health problem, and it has become the second leading cause of death in Western countries [133]. Surgery, antiblastic chemotherapy (CT), target therapy (TT), immunotherapy and radiotherapy (RT) are the most common treatments for cancer. Antiblastic CT, TT, immunotherapy and RT are used as primary treatment approaches in most cancer patients and play vital roles in cancer treatment because many patients are diagnosed at advanced stages, compromising the option of surgery treatment [134]. However, more tumors have become resistant to CT, TT, immunotherapy and RT, which has become a major problem in cancer therapy. Furthermore, complications emerge when cancer cells develop chemoresistance and radioresistance via multiple mechanisms, and the CT agents and RT often cause adverse events [135]. Therefore, we need to identify a new strategy or new therapeutic agent that can overcome chemoresistance and radioresistance. A large number of natural products are considered to be effective anticancer drugs [136]. Most of them are found in fruit and vegetables, such as polyphenols and resveratrol [137]. These compounds have multiple effects in several chronic disease treatments, including cancer [136,137]. Resveratrol has been shown to regulate cellular growth in many human cancer cell lines [138]. Especially, its role in inducing growth inhibition, cell cycle arrest and apoptosis suggested the possibility to use resveratrol to prevent cancer (Figure 6) [139,140]. A number of studies highlighted that this cell-growth modulating effect is both dose- and time-dependent [141,142]. However, the exact mechanism of these actions is not fully understood [136,142]. A high concentration of radical oxygen species (ROS) is found in cancer cells. ROS are a cornerstone in cancer onset and progression through the promotion of cell growth, immortalization and aberrant inhibition of locomotion and proliferation [143]. Resveratrol carries out its antioxidant properties through several mechanisms [143,144]. Especially, resveratrol modulates antioxidant enzymes such as superoxide dismutase, catalase and glutathione peroxidase [144]. Therefore, it induces cancer cell apoptosis by accumulation of hydrogen peroxide [144]. Resveratrol can also downregulate hypoxia-inducible factor-1α (HIF-1α) accumulation, leading to the suppression of fluorodeoxyglucose (F-FDG) uptake from metabolically active cells and glycolysis [54,145].

**Figure 7:**
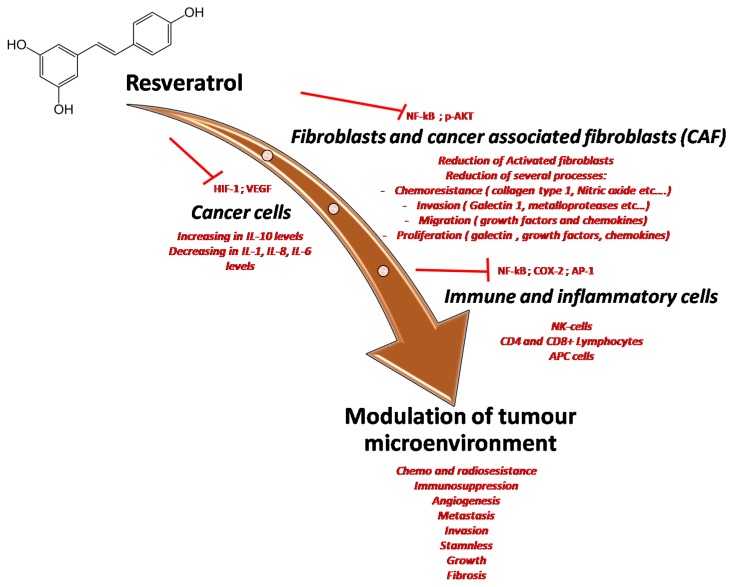
Multiple effects of resveratrol on the tumor microenvironment through the inhibition of many pro-inflammatory mediators and cytokines involved in cancer cell survival, in cancer-associated fibroblast activation and modulation of immune cells resident in tumor tissue.

The tumor microenvironment (TME) is defined as the totality of normal cells, molecules and blood vessels surrounding and feeding each cancer cell [146]. TME cells are stromal cells, immune-inflammatory cells and vascular endothelial cells. Their interactions with each other and with the cancer cells through the secretion of cytokines and chemokines play a fundamental role in tumor initiation and progression [146]. As a matter of fact, a healthy TME protects against tumorigenesis [147]. On the contrary, disorders of the TME increase the levels of inflammatory cytokines, such as tumor necrosis factor α (TNF-α), promoting tumorigenesis through cell immortalization [147]. Moreover, TME governs chemo- and radio-resistance [148]. Therefore, targeting TME to defy cancer could be a winning strategy, both because of its involvement in tumorigenesis and the fact that it is less prone to mutate and rapidly cause metastatic events [148]. Cancer is characterized by a chronic inflammatory state, derived from the activation of both the immune and adaptive immune system cells present within the TME [146]. Thanks to its action on antioxidant enzymes and HIF-1α, among other effects, resveratrol has been proposed as a possible agent able to downregulate chronic inflammation in TME (Figure 7) [149]. In fact, resveratrol downregulates the production of inflammatory cytokines and increases the release of anti-inflammatory mediators by inhibiting AP-1 and NF-κB [150]. TME is a hypoxic environment, created by a rapid cancer cell growth and dysregulation of angiogenesis [149]. HIF-1α, a transcription factor regulating the expression of more than a hundred genes, is overly expressed during hypoxia, promoting more aggressive behaviors of the cancer such as chemo-resistance and metastatic spread [54,149]. As previously mentioned, resveratrol is able to modulate the expression of HIF-1α, thus reducing the fitness of TME to the expansion of the tumor [149]. Modulation of HIF-1α leads to another important effect of resveratrol as an anti-tumor agent. Through downregulation of HIF-1α and increased expression of thrombospondin-1, resveratrol acts as an anti-angiogenic drug [149]. Resveratrol was also demonstrated to be able to interfere with the activation of some immune cells through the inhibition of the proliferation of both T and B lymphocytes and the downregulation of the expression of cluster of differentiation CD28 and CD80 on T-lymphocytes and macrophages, respectively [151]. A particular subset of macrophages, named tumor-associated macrophages (TAMs), are activated by cancer cells and promote the production of inflammatory cytokines through a STAT3 pathway. Resveratrol is able to decrease STAT3 activation in cancer [152]. In addition, resveratrol modulates the PI3K pathway in cancer cells, which downregulates CD8+ cytotoxic T cell activity. Therefore, by inhibiting PI3K, resveratrol enhances anti-cancer immunity [75].

## 7. Conclusion and Perspectives

An increasing number of clinical trials are supporting the benefits of resveratrol in the treatment of chronic diseases, although its use in clinical practice remains very limited. In order to unlock the therapeutic potential of resveratrol and its derivatives, further investigations are warranted aiming to enhance its pharmacokinetic profile through testing new, innovative formulations. Moreover, future clinical trials should investigate the drug interaction between anticancer agents, resveratrol and enzymes activity, particularly focusing on CYP2C9, CYP2C19 (phase I enzyme), GSTP1 and UGT (phase II enzyme) [153]. Promisingly, evaluation of individual metabolic profiles by pharmacogenetic tests should be accurate to perform a personalized therapy in cancer “frail patients” (i.e., HIV) where supplementation of resveratrol allows the reduction of antineoplastic drug dosages [154,155].

## Figures and Tables

**Table 1:** Resveratrol main effects.

Effect	Pathway	Reference
Antioxidant	Copper-chelant	[44,45,49]
	Activation p38-MAPK	[52]
	Inhibition QR2	[48]
	Up-regulation SOD	[47]
Anti-Inflammatory	Inhibition COX_1_	[55,56,58]
	Inhibition COX_2_	[55,56,58]
	Blocked TxA_2_	[57,58]
	Blocked MAPK	[57,58]
	Activation Sirt-1→↑TLR4/NF-κB/STAT; ↓TNF-α/IL-6/IL-1β	[63,64]
Vasodilatation	Activation K^+^ channel Ca^2+^→↑eNOS, ↑eNOS	[60,61,62]
Metabolic Disorder (CVD/T2DM/Obesity)	Activation Sirt-1→ Activation PGC-1_α_→↓plasma levels glucose & glycated	[68,69,70]
	Activation AMPK	[68,69,70]
Cancer	Inhibition Sirt-1/PTEN/PI3K/AKT	[75,76,77,78,79,80]
	Upregulation p21/p53	[81,82,83]
	Inhibition AMPK/YAP	[54,84,85,86]
	Inhibition NF-κB/STAT3	[78]
	Downregulation HIF-1α	[54]

**Table 2:** Metabolic effects and cancer–drug interactions.

Metabolic Enzyme (Direct Effect)	Documented Interaction. Plasma Levels: (H) Higher (L) Lower Dosage	Potential Cancer Drug Interaction	Pharmacogenomic Test	Final Consideration [Reference]
CYP1A1 (Inhibition)	Testosterone # Caffeine (in human) Phenacetin	Bendamustine	CYP1A2*F 5′UTR -163C>A rs7625551	[123]
CYP1B1 (Inhibition)	(L) Cathecol estrogens (anti-breast cancer activity of resveratrol) #	ND	ND	1 g/day for 12 weeks had a favorable effect in post-menopausal women [124]
CYP2B6 (Inhibition)	ND	Cyclophosphamide	ND	[127]
CYP2C9 (Inhibition)	(H) Warfarin #	ND	CYP2C9*2 430C>T R144C. rs1799853 CYP2c9*3 1075A>C I359L rs1057910	1 g daily resveratrol inhibited CYP2C9 by 2.71-fold [126]
CYP2D6 (Low inhibition)	(L) dextromethorphan $	Tamoxifen	CYP2D6*3 2459delA frameshift rs35742686 CYP2D6*4 1846G>A splicing rs3892097 CYP2D6*10 100C>T P35S rs1065852 CYP2D6*XN copy number variation	Probable low activation of tamoxifen in the active metabolite endoxifen [122]
CYP2C19 (Moderate Inhibition)	(H) Pantoprazole $	ND	CYP2C19*17 -806C>T 5′UTR rs12248560	[121]
CYP3A4 (Inhibition)	(H) Nicardipine $ (H) diltiazem $ (H) Carbamazepine $	Imatinib Docetaxel	CYP3A4*22 15389C>T 5”UTR rs35599367	[125,128]
GST (Induction)	(L) nitrosamines and polycyclic aromatic Hydrocarbon #	(H) platin derivates	*GSTP1* Iso105Val	[129]
NQO1 (Induction)	(L) Estrogens by inactivation by catechol-O-methyl transferase #	ND	ND	[130,131]

# evaluated in a murine model; $ evaluated in a cell model; ND no data available.
